# Transcriptional profiling unravels potential metabolic activities of the olive leaf non-glandular trichome

**DOI:** 10.3389/fpls.2015.00633

**Published:** 2015-08-13

**Authors:** Konstantinos Koudounas, Maria E. Manioudaki, Anna Kourti, Georgios Banilas, Polydefkis Hatzopoulos

**Affiliations:** ^1^Department of Biotechnology, Agricultural University of AthensAthens, Greece; ^2^Department of Horticultural Genetics and Biotechnology, Mediterranean Agronomic Institute of ChaniaCrete, Greece; ^3^Department of Oenology and Beverage Technology, Technological Educational Institute of AthensAthens, Greece

**Keywords:** multicellular trichomes, next-generation sequencing, non-glandular trichomes, *Olea europaea*, transcriptome

## Abstract

The olive leaf trichomes are multicellular peltate hairs densely distributed mainly at the lower leaf epidermis. Although, non-glandular, they have gained much attention since they significantly contribute to abiotic and biotic stress tolerance of olive leaves. The exact mechanisms by which olive trichomes achieve these goals are not fully understood. They could act as mechanical barrier but they also accumulate high amounts of flavonoids among other secondary metabolites. However, little is currently known about the exact compounds they produce and the respective metabolic pathways. Here we present the first EST analysis from olive leaf trichomes by using 454-pyrosequencing. A total of 5368 unigenes were identified out of 7258 high quality reads with an average length of 262 bp. Blast search revealed that 27.5% of them had high homologies to known proteins. By using Blast2GO, 1079 unigenes (20.1%) were assigned at least one Gene Ontology (GO) term. Most of the genes were involved in cellular and metabolic processes and in binding functions followed by catalytic activity. A total of 521 transcripts were mapped to 67 KEGG pathways. Olive trichomes represent a tissue of highly unique transcriptome as per the genes involved in developmental processes and the secondary metabolism. The results indicate that mature olive trichomes are trancriptionally active, mainly through the potential production of enzymes that contribute to phenolic compounds with important roles in biotic and abiotic stress responses.

## Introduction

Trichomes are epidermal appendages on leaves or other vegetative and reproductive aerial organs. They are widespread in the plant kingdom, while their morphology varies substantially among tissue and species. Functionally, they are categorized into two major groups: glandular trichomes and non-glandular trichomes, also referred to as “simple hairs”. Glandular trichomes synthesize, accumulate, and often secrete a variety of secondary metabolites, such as terpenes, fatty acid derivatives, alkaloids or defense proteins, most of which protect the plant from abiotic and biotic stresses (Eisner et al., 1998; Iijima et al., 2004; Schilmiller et al., 2008). From the biotechnological view-point, many of these compounds are of great interest for food and pharmaceutical industries. Significant efforts have been made to elucidate their biosynthetic pathways and to manipulate trichomes as “chemical factories” for high-yield production of valuable metabolites. Like glandular trichomes, non-glandular hairs vary in shape, size, and structure, being unicellular or multicellular, branched or unbranched. Principally, they are supposed to prevent attacks from herbivores and they may also control leaf temperature and water loss or serve as a protective layer against solar radiation (Wagner et al., 2004; Dalin et al., 2008). The non-glandular trichomes of Arabidopsis have been used as model to study plant cell differentiation (Hülskamp, 2004). A recent study showed that Arabidopsis trichomes are able to produce glucosinolates, suggesting that non-glandular trichomes, like glandular trichomes, may have an active secondary metabolism (Frerigmann et al., 2012). Non-glandular hairs may also have economic importance. A high value typical example is the cotton (*Gossypium hirsutum*) fiber used in textile industry (Jakoby et al., 2008).

**Figure 1:**
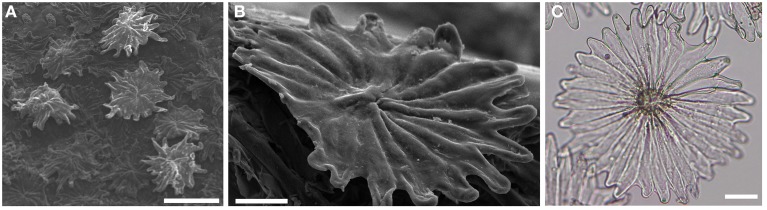
*****Olea europaea*** abaxial trichomes as visualized with scanning electron microscopy (A,B) or light microscopy (C)**. Scale bars: 100 μm **(A)** or 20 μm **(B,C)**.

Olive (*Olea europaea*) leaves are covered by peltate non-glandular trichomes, consisting of a short stalk embedded into epidermal cells and a multicellular shield-like top (Figure 1) (Fahn, 1986; Grammatikopoulos et al., 1994). Young olive leaves have trichomes on both sides. As the olive leaf expands, the trichome density is gradually reduced at the upper (adaxial) epidermis. Thus, in mature leaves trichomes densely cover only the lower epidermis (abaxial surface). During the early stages of trichome development, olive trichome cells display a typical meristematic appearance. They have thin cell walls and numerous small vacuoles, while in mature hairs the cell wall of the stalk becomes fully cutinized, thus preventing water flow into the trichomes (Fahn, 1986).

Olive is a xeromorphic species with hypostomatic leaves hence the dense trichome at the abaxial surface has been proposed to be associated with adaptation to arid conditions via the control of respiration and temperature regulation (Fahn, 1986). Furthermore, olive hairs contain large amounts of UV-B absorbing compounds, such as various flavonoids, that may prevent stomatal closure caused by UV-B radiation (Grammatikopoulos et al., 1994). Besides their protective role as mechanical barriers, olive trichomes could be considered as a highly specialized storage tissue of defense-related phenolic compounds.

Although the olive genome sequence is not yet available, significant progress has been achieved in elucidating various metabolic pathways and identifying key regulatory genes of this important fruit crop. By using next generation sequencing (NGS) technology, transcriptomes of various olive organs were defined and a number of genes involved in lipid or phenolic metabolism in olive fruits were determined and assessed (Alagna et al., 2009, 2012; Muñoz-Mérida et al., 2013). Despite the progress on olive transcriptomics, a critical question is whether the olive trichome, as a highly differentiated plant cell type, is metabolically active and whether specialized biosynthetic pathways are operational. With the advent of the NGS technology, abundant sequence information has been obtained regarding expressed genes in trichomes of several species. However, apart from the non-glandular hairs of the model plant Arabidopsis and those of cotton, research so far has been mainly focused on glandular trichomes. Here we report an olive trichome EST analysis by using the 454-pyrosequencing technology. This is the first attempt to resolve potential metabolic functions and to identify key enzymes that could be implicated in the primary and secondary metabolism of the olive trichomes and genes engaged in differentiation process.

## Materials and methods

### Plant material and cDNA synthesis

Young fully expanded leaves were collected from *Olea europaea* L. cv. “Koroneiki” grown in a natural environment at the Agricultural University of Athens. Trichomes were dissected out from the abaxial leaf side by careful scraping the surface with razor blades and immediately frozen in liquid nitrogen. Samples of trichomes were examined under a light microscope (Olympus BX50) to observe any contamination from adjacent tissues and photographed. Leaf trichomes were also observed under the JSM-6510 JEOL scanning electron microscope (SEM). Total RNA was isolated from trichome cells following a phenol:chloroform extraction procedure as described previously (Haralampidis et al., 1998). After treatment with RNase-free DNAse I (Promega), total RNA concentration and purity was determined spectrophotometrically and checked for degradation by agarose gel electrophoresis. cDNA was synthesized using 100 ng total RNA as starting material and the SMART cDNA Synthesis technology (SMART™ cDNA Library Construction Kit, Clontech, USA), which allows for selective transcription and amplification of polyadenylated mRNA, following the manufacturer's instructions with the following minor modifications. First-strand cDNA synthesis was performed using the PyroRT primer [5′-ACC AGGTCACTCGAGGACATGTTTTTTTCTTTTTTTTTT (N-1)(N)-3′, where N-1 = A, G, or C; N = A, G, C, or T] and the Superscript™ II RNase H- Reverse Transcriptase (Invitrogen). cDNAs were further amplified by long distance PCR (LD-PCR), as described in the SMART™ cDNA Library Construction Kit, by applying 28 PCR cycles. LD-PCR was performed with the provided SMART 5′ PCR forward primer (5′-AAGCAGTGGTATCAA CGCAGAGT-3′) and the reverse primer PyroREV [5′-ACC AGGTCACTCGAGGACATGTTTTGTTCTTGTTGTTTT(N-1)(N) -3′] that introduces “point mutations” in the polyA tail to facilitate sequencing through this repetitive region. To remove low-molecular weight cDNA fragments, size fractionation was carried out using CHROMA SPIN-400 columns according to the manufacturer's protocol. After size determination by agarose gel electrophoresis, the fractions containing cDNAs with an average length over 500 bp were pooled and the concentration was determined using the NanoDrop ND-1000 spectrophotometer (NanoDrop Technologies, USA).

### 454 Pyrosequencing

Approximately, 14 μg of the cDNA population was sheared by nebulization and the sample was adaptor-ligated and amplified by emulsion PCR for sequencing, following protocols for the Genome Sequencer 454 GS FLX system (Roche Diagnostic). Lastly, 125,000 of the enriched beads were loaded onto a picotiter plate (1/16 was used) and sequenced with the 454 GS FLX system at the Institute of Marine Biology and Genetics (IMBG), Crete, Greece. Data obtained from the pyrosequencing analysis have been deposited in the Short Read Archive (SRA) database (http://www.ncbi.nlm.nih.gov/sra) under the accession number SRS897386.

### Clustering, annotation and functional characterization of unigenes

Primer sequences were trimmed from reads by using the cutadapt (v.0.9) tool. Highly similar and overlapping sequences were clustered using the CD-HIT-EST package (http://weizhong-lab.ucsd.edu/cd-hit/) using a 95% identity threshold and a minimum length of 100 bp. Algorithm parameters were set so as to compare both strands and assign the clustered sequences to the most similar cluster, rather than the first cluster that meets the threshold. The resulting sequences (unigenes) were aligned with NCBI non-redundant protein database (Nr) using blastx (blast 2.2.25+ version) with a cut-off *E*-value of 1.0E-6. Blastx results were mapped to retrieve Gene Ontology (GO) terms by using Blast2GO. Blast2GO was further used to assign biological functions, cellular components, and cellular processes to transcripts. Mapped sequences were annotated against the Kyoto Encyclopedia of Genes and Genomes (KEGG) database (http://www.genome.jp/kegg/) to obtain enzyme commission (EC) numbers. The EC numbers were then mapped to the KEGG biochemical pathways to obtain KEGG Pathway-Maps.

### Semi-quantitative RT-PCR analysis

The first-strand cDNA was synthesized from 1 μg total RNA from olive leaf trichomes or leaves after trichome removal using a reverse transcription oligo-dT primer. RT-PCR analysis of selected unigenes was applied to verify gene expression in trichomes and compare transcript abundances with trichome-minus leaves. Actin and a small subunit of Rubisco (*OeSRB*) were used as internal control. PCR amplification was performed using gene-specific primers (Table S1).

## Results and discussion

### EST analysis and annotation of transcripts

**Figure 2:**
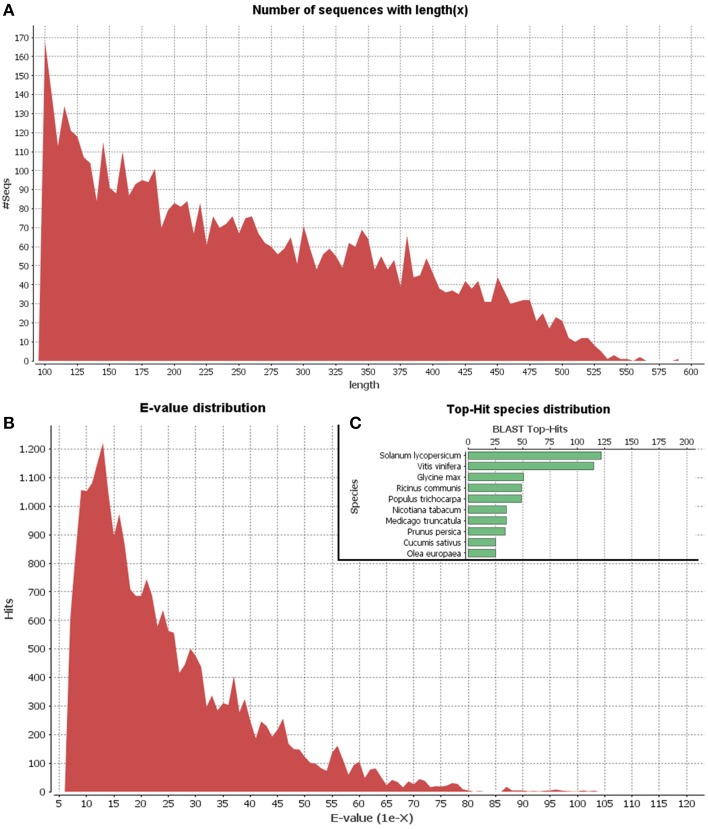
**Length distribution of unigenes (A)**. *E*-value distribution of Blastx hits for unigenes with a cutoff *E*-value of 1E-6 **(B)**. Top 10 species with Blast Top-Hits **(C)**.

Since the olive genome has not been sequenced as yet and the olive trichome represents a highly specialized tissue, we applied 454-pyrosequencing which results in long read lengths. A cDNA library of the olive trichome was prepared and sequenced using the GS FLX platform generating a total of 13,059 sequence reads of about 2.5 Mb total length. Comparable results were obtained using pyrosequencing analysis in basal endosperm transfer cells in maize (Xiong et al., 2011). Following, processing of the raw data to trim oligonucleotide sequences and remove adapters, a total of 7258 high quality reads with an average length of 262 bp were obtained. Their minimum and maximum lengths were 100 bp and 593 bp, respectively. Using CD-HIT-EST, 5368 non-redundant EST clusters were generated. The vast majority of the clusters (4599) were identified as singletons (having only one non-redundant member) while each of the rest 769 clusters contained 2-67 ESTs. Singletons and ESTs from each cluster are hereafter collectively referred to as unigenes. The length distribution of unigenes is shown in Figure 2A. Blast searches of unigene sequences against the NCBI Nr protein database revealed that 1481 out of the total 5368 unigenes (27.5%) were homologous to known proteins at the cut-off *E*-value of 1.0E-6. The *E*-value distribution of the top hits in the Nr database revealed that the respective homologies were highly significant for the majority of the mapped sequences (less than 1.0E-10) (Figure 2B). The top-three hits were obtained against *Solanum lycopersicum, Vitis vinifera*, and *Glycine max* (Figure 2C). Similar, results were obtained in a recent transcriptome analysis in various olive organs (Muñoz-Mérida et al., 2013).

**Figure 3:**
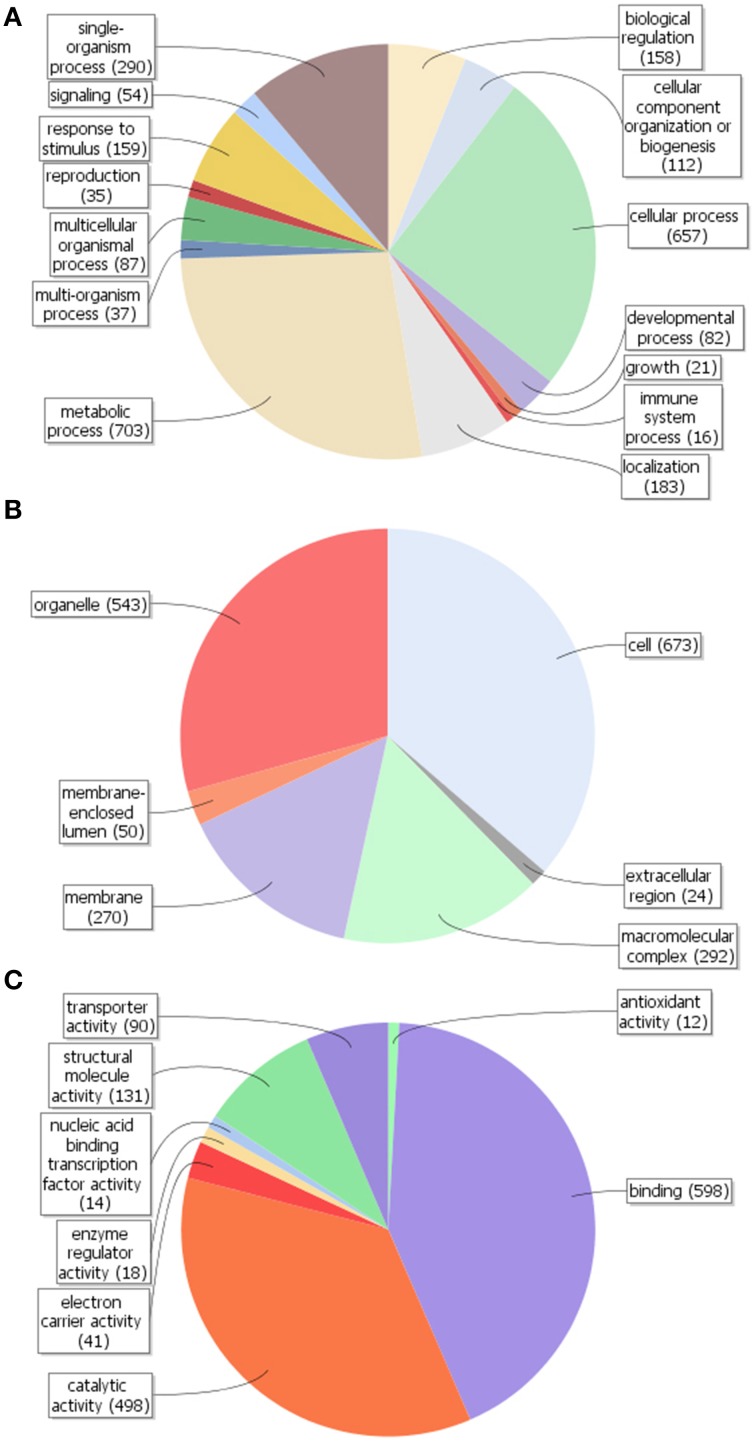
**Gene ontology (GO) assignment (Level 2 GO terms) for the unigenes of ***Olea europaea*** trichomes**. Results are summarized for the three main GO categories: **(A)** biological process, **(B)** cellular component, and **(C)** molecular function. The numbers in parentheses represent the unigenes annotated with the respective GO term.

By using Blast2GO *in silico* analysis, GO terms were assigned to the unigenes previously annotated by the Nr database. A total of 1079 unigenes (20.1%) were assigned at least one GO term (Figure S1). Comparison of GO classification within each domain revealed that most of the unigenes within the biological process category were putatively involved in cellular (GO:0009987) and metabolic (GO:0008152) processes, followed by single-organism process (GO:0044699) (Figure 3A). The majority of gene products within the cellular component category were predicted to be localized in the cell part (GO:0005623), followed by the organelles (GO:0043226) and in macromolecular complexes (GO:0032991) (Figure 3B). As per the molecular functionality category, most genes were involved in binding (GO:0005488), catalytic (GO:0003824) and structural molecule (GO:0005198) activities (Figure 3C). It is therefore likely that olive trichomes remain metabolically active in fully expanded leaves corroborating the results and the functional annotations obtained from non-glandular hairs studied so far (Wagner et al., 2004).

To further identify putative functions of the olive trichome, unigenes were annotated against the KEGG database and EC numbers for enzyme coding genes were mapped to the KEGG reference metabolic pathways. Overall, 521 transcripts were mapped to 67 KEGG pathways, the most represented of which are shown in Table 1. Among them, a number of unigenes were potentially involved in trichome development and differentiation, response to biotic or abiotic stress, and biosynthesis of secondary metabolites. Further, a number of unigenes were assigned to diverse primary metabolic pathways indicative of the pluripotent metabolism that possibly occurs within the trichome cells.

**Table 1:** **The top 15 most represented KEGG pathways**.

**KEGG pathway**	**No. of unigenes**	**No. of enzymes**
Oxidative phosphorylation	38	6
Purine metabolism	27	10
Starch and sucrose metabolism	15	10
Glycolysis/Gluconeogenesis	14	6
Carbon fixation	13	6
Pyruvate metabolism	12	7
Arginine and proline metabolism	9	7
Tryptophan metabolism	9	3
Fatty acid degradation	9	3
Pyrimidine metabolism	8	2
Glutathione metabolism	7	6
Phenylpropanoid biosynthesis	7	3
Glyoxylate and dicarboxylate metabolism	6	5
Pentose and glucuronate interconversions	6	4
Alanine, aspartate, and glutamate metabolism	5	5

### Genes involved in carbohydrate and energy metabolism

The majority of transcripts were implicated in the category of carbohydrate metabolism, mainly in glycolysis/gluconeogenesis, starch and sucrose or pyruvate metabolism (Table 1). It is likely that olive trichomes metabolize high amounts of supplemented carbon substrates (e.g., sucrose, glucose) for energy demands or biosynthesis of structural polysaccharides, since they are considered photosynthetically inactive. Supporting this, we found homologs of sucrose synthase (EC: 2.4.1.13), phosphate transporter 1;5 (Pht1;5, AT2G32830) and mannitol transporter, which are crucial in heterotrophic tissues for sucrose partitioning, phosphate, and mannitol mobility, respectively (Table S2) (Sturm and Tang, 1999; Conde et al., 2007; Nagarajan et al., 2011; Jin et al., 2014). Herein we identified 6 unigenes encoding enzymes involved in glycolysis/gluconeogenesis. Similarly, transcriptomic analysis of Arabidopsis trichome classified 9 enzymes involved in the gluconeogenesis pathway, required for cell wall biosynthesis, a process that is quite active during trichome growth (Jakoby et al., 2008).

Key enzymes active in diverse biochemical pathways but mainly in oxidative phosphorylation and carbon fixation were identified in the KEGG category of energy metabolism. In addition several transcripts analyzed in this study encode proteins implicated in defense responses. Among them, 6 are involved in glutathione metabolism. Apart from its various physiologically roles in plants (Herschbach et al., 1998), glutathione and its reduced form have been considered to play important roles against biotic and abiotic stresses. A total 300-fold higher content of glutathione has been reported in Arabidopsis trichomes than in epidermal cells and attributed to detoxification processes (Gutierrez-Alcala et al., 2000).

### Genes with putative implication in amino acid metabolism

Genes engaged in amino acid biosynthetic pathways especially in arginine and proline metabolism constituted the second most represented KEGG category, (Table 1). Proline is well-known to accumulate under environmental constraints and plays central roles, although not yet fully understood, in abiotic stress tolerance of plants. L-arginine serves as an important nitrogen reserve and recycling. It is also essential for plant adaptation to environmental disturbances, including abiotic stress response, through the production of chemical compounds such as nitric oxide (Tuteja and Sopory, 2008). Considering that we identified several transcripts encoding ribosomal proteins along with factors involved in initiation of transcription or translation (Table S2), it is plausible that the protein synthesis machinery in olive trichomes is very active.

### Genes with putative functions in trichome development

**Figure 4:**
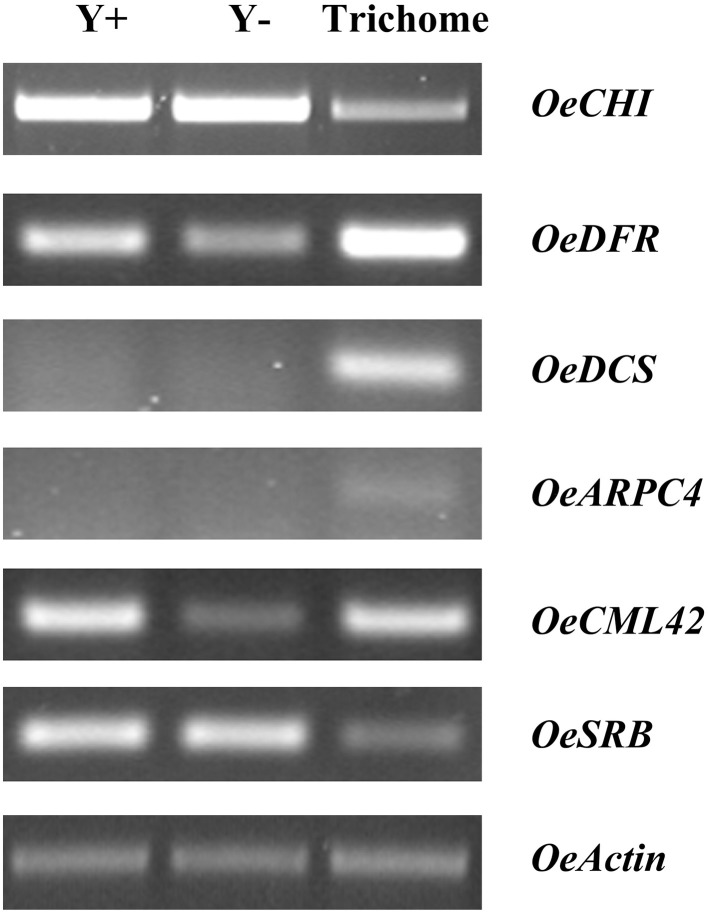
**Semi-quantitative RT-PCR analysis for selected ESTs among young leaves (Y+), dehaired young leaves (Y−) and trichomes**. *OeCHI*, chalcone-flavanone isomerase; *OeDFR*, dihydroflavonol 4-reductase; *OeDCS*, 6′-deoxychalcone synthase; *OeARPC4*, actin related protein 2/3 complex, subunit 4; *OeCML42*, calmodulin-like protein 42; *OeSRB*, small subunit of rubisco; *OeActin*, actin.

Even though significant progress toward the exploration of Arabidopsis trichome initiation and development has been made, detailed molecular switches that control signaling hubs are still poorly described (Jakoby et al., 2008). In this study, several ESTs were identified that are related to trichome development and morphology (Table 2), such as a calmodulin-related calcium sensor *CML42* and a homolog of *ARPC4* encoding the 20-kDa subunit (P20-ARC) of the ARP2/3 complex. Semi-quantitative RT-PCR analysis revealed that both genes were expressed at much higher levels in trichomes than in trichome-less leaves (Figure 4). Calmodulin-like proteins (CMLs) are plant-specific sensor relay proteins with various roles in plant development. In Arabidopsis, loss of CML42 (At4g20780) function leads to irregular trichome morphology with increased branching. The gene is also involved in insect herbivory defense and abiotic stress responses (Dobney et al., 2009; Vadassery et al., 2012). The Arp2/3 protein complex initiates actin polymerization, while ARPC4 (At4g14147) is the most critical core subunit controlling its assembly and steady-state levels (Kotchoni et al., 2009). Arabidopsis mutations in different Arp2/3 subunits (referred to as *distorted* mutants) exhibit severe abnormalities in tip growth or root hair development, and irregularities in the leaf trichome shape, since both trichome initiation and branching depend on actin polymerization (El-Assall et al., 2004; Zhang et al., 2005; Kotchoni et al., 2009).

**Table 2:** **Unigenes with potential implication in olive trichome development and morphology**.

**Unigene**	**Annotation**	**GO ID**	**Top blast**	**Accession number**	***E*-value**
GRNLHQF01ANLBE	WD-repeat partial	Trichome morphogenesis	*Silene latifolia*	AAM81257.1	3.23E-10
GRNLHQF01APWEX	Protein translocase subunit chloroplastic-like (AGY1)	Trichome morphogenesis	*Solanum lycopersicum*	XP_004229503.1	1.87E-21
GRNLHQF01AQURN	Calcium binding protein (CML42)	Trichome branching	*Citrus sinensis*	ABK06394.1	7.65E-21
GRNLHQF01ARIT8	Bahd acyltransferase dcr-like (PEL3)	Trichome morphogenesis	*Cucumis sativus*	XP_004140767.1	3.77E-21
GRNLHQF01AHV5N	Actin-related protein 2 3 complex subunit 4-like (ARPC4)	Cell morphogenesis	*Glycine max*	XP_003524942.1	1.08E-46

An olive unigene similar to Arabidopsis *PERMEABLE LEAVES 3* gene (*PEL3*; At5g23940), sharing high similarity with a homolog gene of *Cucumis sativus*, was identified. Mutations in *PEL3* showed phenotypes with occasional tangling of expanding trichomes. PEL3 protein is localized both in the nucleus and the cytoplasm of Arabidopsis trichomes (Marks et al., 2009; Panikashvili et al., 2009). Finally, a unigene similar to Arabidopsis *AGY1* (Albino or Glassy Yellow 1, At4g01800), which encodes a subunit of the translocase subunit secA, was identified. Loss of AGY1 function leads to decreased branching of Arabidopsis trichome. Transcriptional analysis employing the β-glucuronidase (*GUS*) reporter gene showed expression both in the base and the tip of the Arabidopsis trichome, suggesting a prominent role in trichome morphogenesis (Liu et al., 2010).

Interestingly, none of the master regulators of trichome formation in Arabidopsis like *GLABRA 1-3, TRANSPARENT TESTA GLABRA 1* and *2, TRIPTYCHON* and *CAPRICE* were identified in this study. However, a unigene was assigned having the motif EGL3 (ENHANCER OF GLABRA3), a DNA binding/transcription factor (PTHR11514:SF8) (Table S2). This outcome could be attributed to the age of the sampled trichomes as these transcription factors are known to play key roles at early stages of trichome formation.

### Genes for specific response to abiotic and biotic stresses

A number of ESTs for abiotic stress response were found, the most important of which are shown in Table S3. Among them, an olive homolog of the Arabidopsis *ITN1* (At3g12360), putatively involved in salt stress tolerance, was detected (Sakamoto et al., 2008). A unigene showing high homology to the Arabidopsis *HOS15* (high expression of osmotically responsive gene 15, At5g67320) was also identified. *HOS15* encodes a WD-40 chromatin repression complex protein involved in histone deacetylation, which is important for freezing stress tolerance (Chinnusamy and Zhu, 2009). A putative ELIP unigene (early light-inducible protein) was found. The photoprotective function of ELIP on plant leaves against photooxidation has been well-documented (Tzvetkova-Chevolleau et al., 2007).

Two transcripts encoding WRKY transcription factors with potential roles in stress response were identified. One of them encodes a putative abscisic acid responsive element-binding protein 2 (ABF2), which is an ABF subfamily member of bZIP proteins with roles in sugar signaling and drought stress tolerance (Kim et al., 2004; Fujita et al., 2005). The other had high homology to the Arabidopsis WRKY11 (At4g31550), which acts as a negative regulator of basal resistance to the bacterial plant pathogen *Pseudomonas syringae* pv. tomato (Pst) and is involved in the regulation of Pst-induced jasmonic acid-dependent responses (Journot-Catalino et al., 2006). WRKY transcription factors may be also implicated in trichome development (Ülker and Somssich, 2004; Ren et al., 2010).

Other ESTs identified with potential functions in biotic stress response include a putative *pathogenesis-related protein 1* (*PR1*) and a *CCR4-associated factor 1a* (*CAF1a*) gene (Table S3). PR proteins have a well-documented protective role against pathogenic fungi and bacteria (Li et al., 2011), while *CAF1* genes show deadenylation activity over stress-related mRNAs (Liang et al., 2009). Since Arabidopsis transgenic plants over-expressing *AtCAF1a* show elevated expression of PR1 and PR2 and increased resistance to Pst infection (Liang et al., 2009), it is likely that the two genes are coordinately transcribed.

### Secondary metabolism-related genes

Plants produce a wide range of secondary metabolites as a protective strategy against different forms of biotic and abiotic stress. Many unigenes involved in the pathway of phenylpropanoids were found (Table 3; Figure S2), like the phenylalanine ammonia lyase (*PAL*) that catalyzes the first step in phenylpropanoid biosynthesis by the deamination of L-phenylalanine to trans-cinnamic acid. Different phenylpropanoid metabolism pathways may provide an array of secondary metabolites like flavonoids and lignin. Cinnamic acid is also a common precursor of various polyphenols that accumulate at maximal levels in the early stages of olive drupe development (Alagna et al., 2009). Phenylpropenes, a class of phenylpropanoids, have been found in the essential oil of basil peltate glands in basil leaves (Gang et al., 2001). Phenylpropanoids may also have repellent roles in defensive phytoalexin responses to infection and attacks by insects and other herbivores or when triggered by cell wall damage (Ferrer et al., 2008; Denness et al., 2011). Supporting this, occurrence of phenolics in olive trichomes has been reported (Liakopoulos et al., 2006). Flavonoids constitute a large category of secondary metabolites and are abundantly produced by plant trichomes. Within the flavonoid biosynthesis pathway, a transcript encoding 6′-deoxychalcone synthase (*DCS*) was identified (Table 3; Figure S3). The DCS catalyzes the production of isoliquiritigenin (6′-deoxychalcone) from 4-coumaroyl-CoA and 3 malonyl-CoA substrates. We also detected a transcript encoding chalcone-flavanone isomerase (CHI) that catalyzes the isomerisation of chalcone to a flavanone (naringenin). From this point forward the pathway diverges into different biosynthetic branches resulting into various classes of flavonoids (Dao et al., 2011). Another crucial enzyme in the flavonoid pathway, the dihydrokaempferol 4-reductase also known as dihydroflavonol 4-reductase (DFR) was identified. This enzyme catalyzes the reduction of dihydroflavonols to leucoanthocyanins. It is a rate limiting enzyme in the biosynthesis of proanthocyanidins, anthocyanins, and other flavonoids important to plant cellular homeostasis and of great interest in human nutrition and medicine (Peters and Constabel, 2002; Huang et al., 2012).

**Table 3:** **Key enzymes mapped to the KEGG secondary metabolism biosynthesis pathways**.

**Unigene**	**EC number**	**Enzyme name**	**Pathway**	**Top blast**	**Accession number**	***E*-value**
**TERPENOID BIOSYNTHESIS**						
GRNLHQF01AK5VI	2.7.1.148	4-(cytidine 5′-diphospho)-2-C-methyl-D-erythritol kinase	Terpenoid backbone biosynthesis	*Vitis vinifera*	XP_002267319	1.89E-15
GRNLHQF01AMC3M	1.1.1.267	1-deoxy-D-xylulose 5-phosphate reductoisomerase	Terpenoid backbone biosynthesis	*Rauvolfia verticillata*	AAY87151	7.92E-21
GRNLHQF01AGOD3	1.17.7.1	4-hydroxy-3-methylbut-2-en-1-yl diphosphate synthase	Terpenoid backbone biosynthesis	*Nicotiana langsdorffii x Nicotiana sanderae*	ABV02021	3.81E-54
**FLAVONOID AND ANTHOCYANIN BIOSYNTHESIS**						
GRNLHQF01AKXK3	5.5.1.6	Chalcone-flavanone isomerase	Flavonoid biosynthesis	*Glycine max*	NP_001242041	8.81E-12
GRNLHQF01AKSGT	1.1.1.219	Dihydroflavonol 4-reductase	Flavonoid biosynthesis	*Solanum lycopersicum*	ABR15768.1	3.33E-65
GRNLHQF01AFCBP	2.3.1.170	6′-deoxychalcone synthase	Flavonoid biosynthesis	*Solanum lycopersicum*	XP_004236903.1	3.71E-34
GRNLHQF01ANJ22	2.4.1.115	Anthocyanidin 3-O-glucosyltransferase	Anthocyanin biosynthesis	*Vitis vinifera*	XP_002269179.1	2.43E-11
**PHENYLPROPANOID BIOSYNTHESIS**						
GRNLHQF01AIVKS	1.2.1.44	Cinnamoyl-CoA reductase	Phenylpropanoid biosynthesis	*Paulownia sp. ZKC-2008*	ACD13265.1	2.28E-49
GRNLHQF01AR7QT	1.1.1.195	Cinnamyl alcohol dehydrogenase	Phenylpropanoid biosynthesis	*Solanum lycopersicum*	ABR15768.1	6.06E-26
GRNLHQF01APWF3	4.3.1.24	Phenylalanine ammonia-lyase	Phenylpropanoid biosynthesis	*Digitalis lanata*	O23924.1	9.71E-22

Semi-quantitative PCR analysis showed that *OeCHI* was expressed in trichomes, although its expression was higher in leaves. It is known that CHI is evenly distributed in leaf epidermal and parenchyma tissues of *Pisum sativum* (Hrazdina et al., 1982). As opposed, the levels of mRNA accumulation for both *OeDCS* and *OeDFR* were much higher in trichomes than in trichome-less leaves (Figure 4) indicative of alternative biochemical routes between olive trichomes and leaf cells. Quantitative and qualitative differences in flavonoid accumulation among leaf lamina and trichomes of olive leaves (Liakopoulos et al., 2006) corroborate our results. Present data suggest that the biosynthesis pathway of flavonoids is most likely active in trichomes of fully-expanded leaves. Thus, additional roles of flavonoids in mature olive trichomes are probable, such as protection against microbial pathogens, insect pests and herbivores (Hichri et al., 2011). Interestingly, a unigene encoding anthocyanidin 3-O-glucosyltransferase, the first enzyme in anthocyanin biosynthesis pathway, was identified (Table 3; Figure S4). Accumulation of anthocyanins in trichomes was recently reported in Gesneriaceae and Lamiaceae species having potential protective roles against UV radiation and pathogens (Zhang et al., 2014).

In addition, phenylpropanoid biosynthesis gives rise to lignin formation. Two important unigenes in the lignin formation pathway were identified, i.e., cinnamoyl-CoA reductase (CCR) that catalyzes the first committed step of lignin-specific branch to produce lignin monomers and cinnamyl-alcohol dehydrogenase (CAD) that acts on the last step in the formation of monolignols. The results strengthen the possibility that the lignin biosynthesis pathway is active in olive trichomes.

Even though, we did not detect any terpene synthases a number of unigenes associated with terpenoids biosynthesis/modifications were identified. We found three out of the total seven unigenes encoding the main enzymes of the plastid-localized 2-C-methyl-D-erythritol 4-phosphate/1-deoxy-D-xylulose 5-phosphate (MEP/DOXP) pathway (Table 3; Figure S5) but none in the cytosolic mevalonate (MVA) pathway (Lichtenthaler, 1999; Newman and Chappell, 1999). It is known that the MEP pathway also supplies precursors for chlorophyll and carotenoids. In olive, both terpenoids and genes involved in terpenoid biosynthesis have been detected in leaf epicuticular waxes and fruits (Bianchi et al., 1992; Alagna et al., 2009) but to our knowledge terpenes have not been found in trichomes, as yet. Glandular trichomes of different plants, like *Salvia divinorum, Nicotiana tabacum*, and *Cistus creticus* (Wang and Wagner, 2003; Siebert, 2004; Falara et al., 2008) or *Ocimum basilicum* and *Mentha piperita* (Iijima et al., 2004) are known to accumulate di- or mono-terpenes, respectively. It should be noted that the contribution of non-glandular trichomes in chemical defense is a matter of debate which has very recently started to be elucidated (Frerigmann et al., 2012).

The dual-partner defense system of β-glucosidase/oleuropein present exclusively in the photosynthetic cells of the leaf (Koudounas et al., 2015) in combination with the (a)biotic resistance-related transcriptome of the trichomes makes the olive leaf a fortified system highly adaptive to harsh environmental conditions and against to biotic stress.

## Conclusions

Using the 454 pyrosequencing technology the first EST dataset from olive leaf trichomes was generated. Although transcriptomes from leaves and other olive organs are available (Muñoz-Mérida et al., 2013), olive was not among the top-hit species in Blast searches of unigene sequences. This suggests that the olive trichome represents a highly specialized tissue, while any possible contamination by adjacent mesophyll tissues was rather negligible. Blast search also revealed that the overall olive trichome transcriptome shares similarities to that of other plant species. Present results show that olive trichomes of fully developed leaves potentially have active metabolism as diverse transcripts of primary metabolism were detected. Secondary metabolism-related genes were identified in different pathways such as flavonoid biosynthesis. These genes show a cell-type specific transcript accumulation necessitating to further study non-glandular trichomes as possible accumulators of high added-value metabolites. Finally, genes associated with responses in (a)biotic stress suggest that olive trichome is an important tissue that fortifies the ecophysiology of the olive tree.

### Conflict of interest statement

The authors declare that the research was conducted in the absence of any commercial or financial relationships that could be construed as a potential conflict of interest.
