# Supplementary material for: Transcriptional profiling unravels potential metabolic activities of the olive leaf non-glandular trichome
Source: Front Plant Sci. 2015 Aug 13;6:633. doi: 10.3389/fpls.2015.00633 (PMC4534801; doi:10.3389/fpls.2015.00633)
Supplement: Supplementary file 1 [file Table1.DOCX]

**Table S1.** List of primers used for Semi-quantitative RT-PCR analysis.

| **Primer** | **Sequence 5’ -> 3’** |
| --- | --- |
| OeActin-F | AAGATGACCCAAATCATGTTTGAGAC |
| OeActin-R | ACGACCTTGATCTTCATGCTGC |
| OeDFR-F | GCAGAACTCTGGATTAGACCAC |
| OeDFR-R | TGATGGAGCCAAGGAGAGACTT |
| OeDCS-F | CTCTTAGTTGTTTGCTGCCACG |
| OeDCS-R | GGATGTGAAGGGAGTTTGGGAA |
| OeARPC4-F | GTTCATCTGCCTGCTTTACC |
| OeARPC4-R | TCGTACTGTAGGCGAACACATC |
| OeCML42-F | GTTAGGATTGCAGGAAGGTGGA |
| OeCML42-R | CCCGAACAAATCATAAGCGC |
| OeCHI-F | CCATCTCGAAGTCGATTCCTAC |
| OeCHI-R | TTTGCTTCAGGGGAAACACCGTG |
| OeSRB-F | CCCGATCTCACTAGGGAACAAC |
| OeSRB-R | AGCTTCCACATGGTCCAGTA |
