# Supplementary material for: Transcriptional profiling unravels potential metabolic activities of the olive leaf non-glandular trichome
Source: Front Plant Sci. 2015 Aug 13;6:633. doi: 10.3389/fpls.2015.00633 (PMC4534801; doi:10.3389/fpls.2015.00633)
Supplement: Supplementary file 3 [file Table3.DOCX]

**Table S3.** Unigenes that mediate abiotic and biotic stress responses.

| **Unigene** | **Annotation** | **GO ID** | **Top Blast** | **Accession number** | **E-value** |
| --- | --- | --- | --- | --- | --- |
| GRNLHQF01AMZCX | early light-inducible protein | **response to UV-B** | *Populus trichocarpa* | EEE89919.1 | 9.00E-46 |
| GRNLHQF01AK5T5 | ras-related protein rab-18 | **response to water deprivation** | *Prunus persica* | EMJ01685.1 | 2.08E-48 |
| GRNLHQF01ANTWB | calmodulin-related protein | **response to cold** | *Solanum lycopersicum* | XP_004231987.1 | 4.3E-35 |
| GRNLHQF01APKTK | wd-40 repeat family protein | **response to cold** | *Arabidopsis thaliana* | BAD94649.1 | 5.51E-25 |
| GRNLHQF01AMXLS | major latex-like protein | **response to biotic stimulus** | *Salvia miltiorrhiza* | ADG43176.1 | 1.67E-23 |
| GRNLHQF01AIU86 | protein tify 10a | **defense response to bacterium** | *Phillyrea latifolia* | CAK18857.1 | 2.23E-15 |
| GRNLHQF01AGSG0 | wrky transcription factor 11 | **defense response to bacterium** | *Ricinus communis* | EEF46802.1 | 4.04E-22 |
| GRNLHQF01AG2UG | ndr1 hin1-like protein 1 | **defense response to virus** | *Fragaria vesca subsp. vesca* | XP_004303497.1 | 1.63E-23 |
| GRNLHQF01AQ82M | ankyrin repeat-containing protein at3g12360-like | **response to salt stress** | *Vitis vinifera* | CBI32660.3 | 4.53E-20 |
| GRNLHQF01AQMR7 | pathogenesis-related protein 1 | **response to**  **stress** | *Vitis hybrid cultivar* | ADN43440.1 | 9.92E-29 |
| GRNLHQF01AI6WG | ribonuclease caf1 | **RNA modification** | *Solanum lycopersicum* | XP_004241682.1 | 6.75E-63 |
| GRNLHQF01AIMTT | metallothionein-like protein | **metal ion binding** | *Olea europaea* | AFP49330.1 | 7.99E-21 |
| GRNLHQF01AMUKA | areb-like protein | **response to**  **water**  **deprivation** | *Solanum lycopersicum* | AAS20434.1 | 1.18E-11 |
