# Supplementary figures and images for: Transcriptional profiling unravels potential metabolic activities of the olive leaf non-glandular trichome
Source: Front Plant Sci. 2015 Aug 13;6:633. doi: 10.3389/fpls.2015.00633 (PMC4534801; doi:10.3389/fpls.2015.00633)

## Slide 1
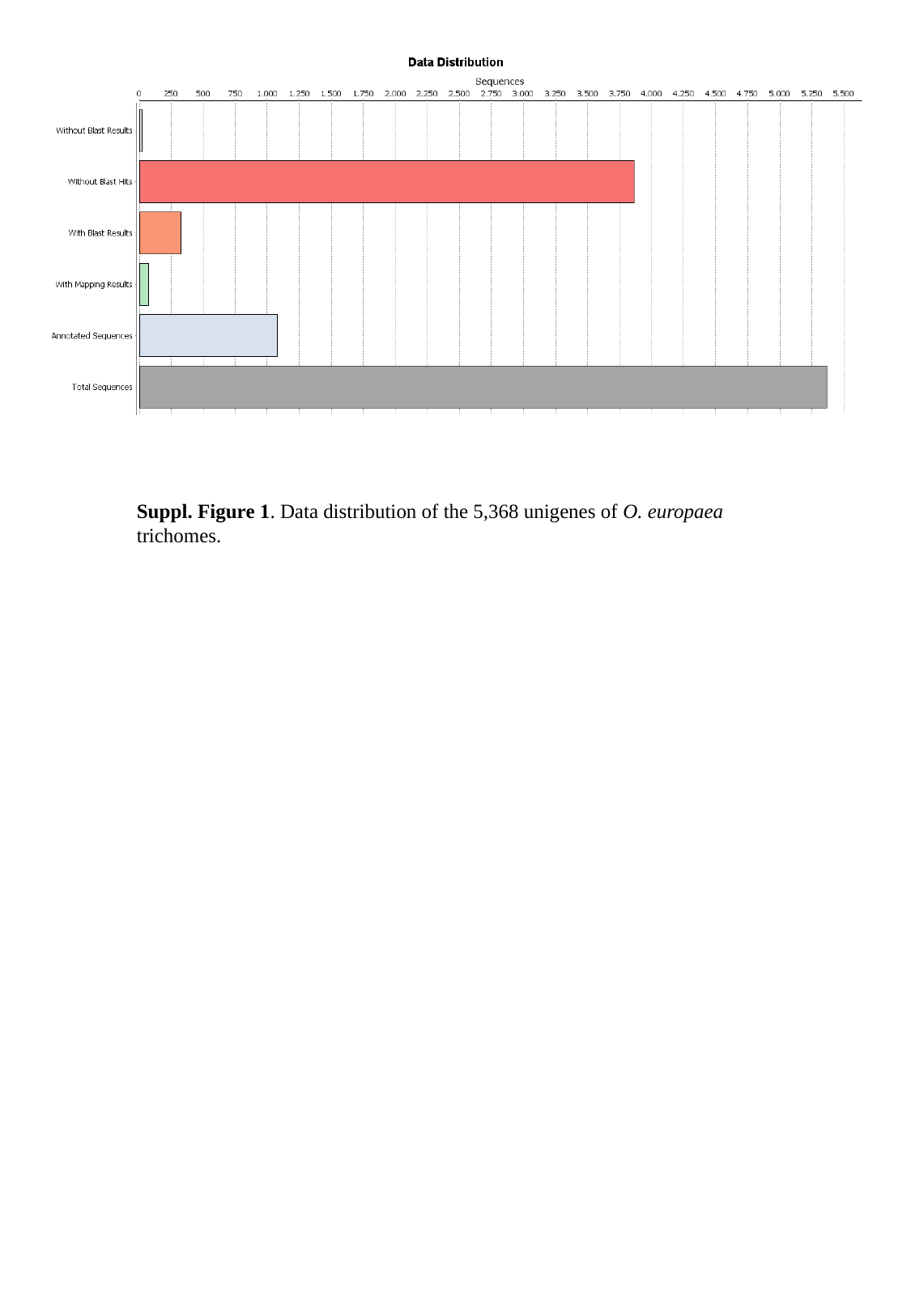

Suppl. Figure 1. Data distribution of the 5,368 unigenes of O. europaea trichomes.

Supplement: Supplementary file 4 [file Presentation1.PPTX]
