# Supplementary material for: Transcriptional profiling unravels potential metabolic activities of the olive leaf non-glandular trichome
Source: Front Plant Sci. 2015 Aug 13;6:633. doi: 10.3389/fpls.2015.00633 (PMC4534801; doi:10.3389/fpls.2015.00633)
Supplement: Supplementary file 5 [file Presentation2.PPTX]

## Slide 1
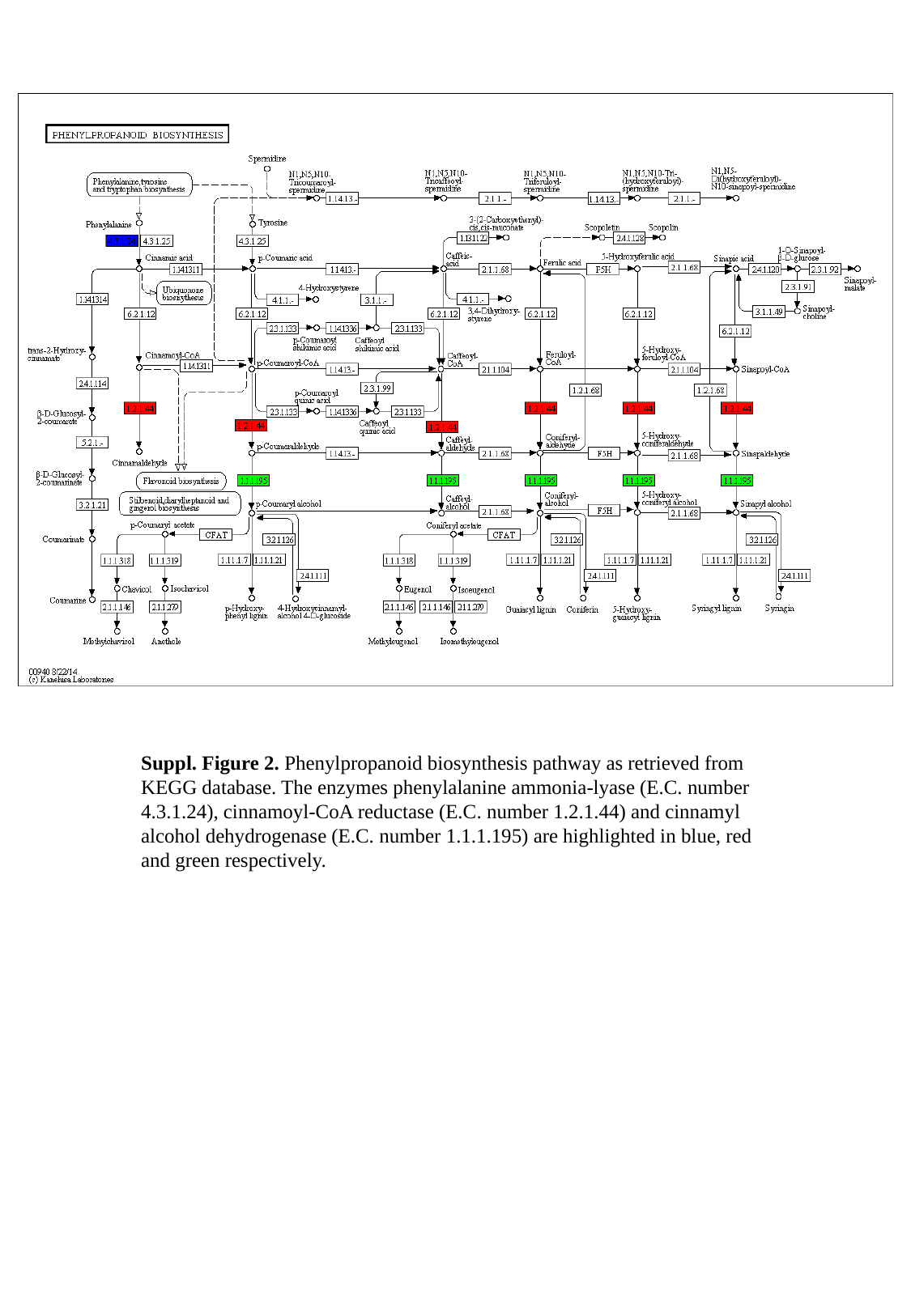

Suppl. Figure 2. Phenylpropanoid biosynthesis pathway as retrieved from KEGG database. The enzymes phenylalanine ammonia-lyase (E.C. number 4.3.1.24), cinnamoyl-CoA reductase (E.C. number 1.2.1.44) and cinnamyl alcohol dehydrogenase (E.C. number 1.1.1.195) are highlighted in blue, red and green respectively.
