# Supplementary material for: Transcriptional profiling unravels potential metabolic activities of the olive leaf non-glandular trichome
Source: Front Plant Sci. 2015 Aug 13;6:633. doi: 10.3389/fpls.2015.00633 (PMC4534801; doi:10.3389/fpls.2015.00633)
Supplement: Supplementary file 6 [file Presentation3.PPTX]

## Slide 1
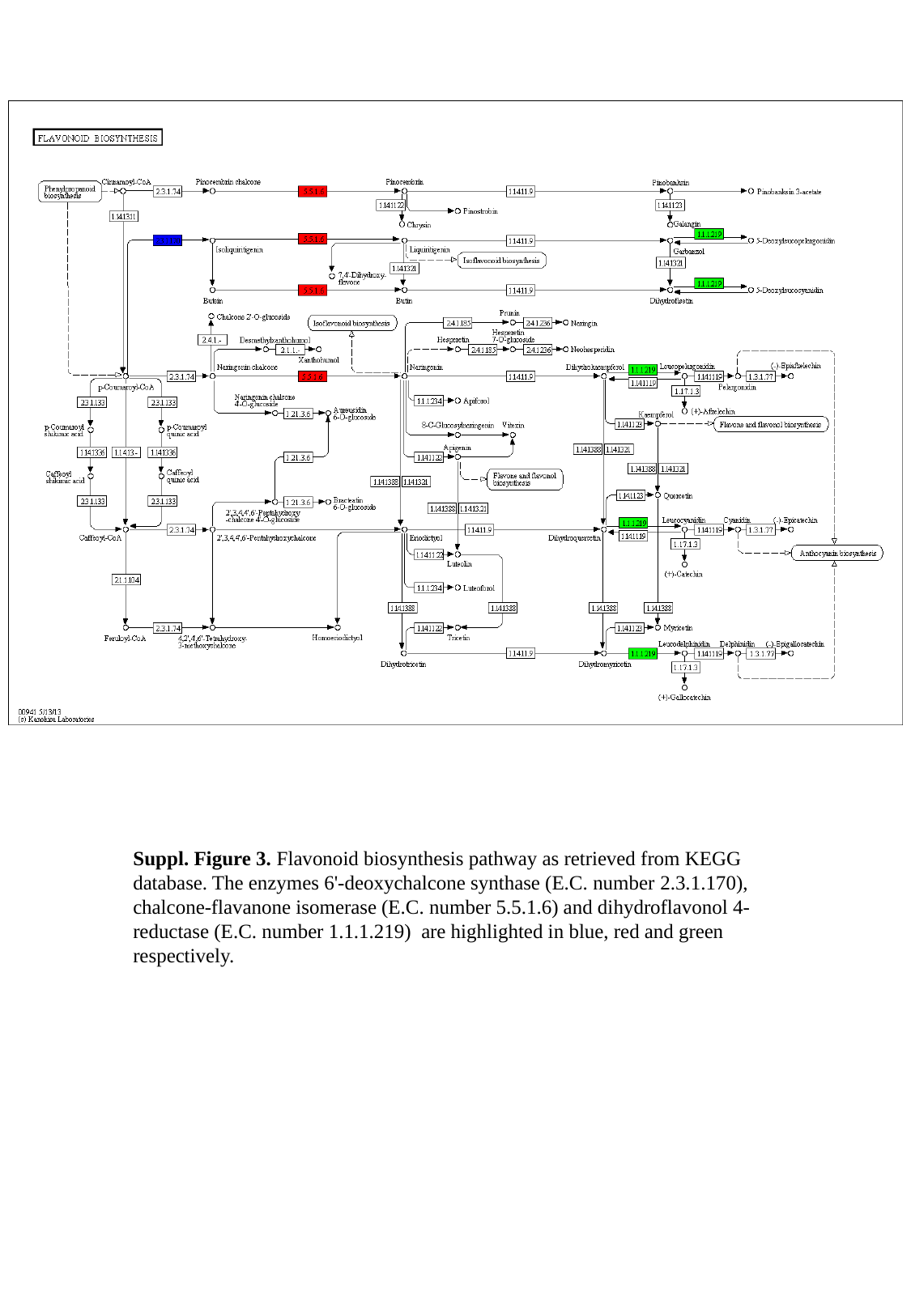

Suppl. Figure 3. Flavonoid biosynthesis pathway as retrieved from KEGG database. The enzymes 6'-deoxychalcone synthase (E.C. number 2.3.1.170), chalcone-flavanone isomerase (E.C. number 5.5.1.6) and dihydroflavonol 4-reductase (E.C. number 1.1.1.219) are highlighted in blue, red and green respectively.
