# Supplementary material for: Transcriptional profiling unravels potential metabolic activities of the olive leaf non-glandular trichome
Source: Front Plant Sci. 2015 Aug 13;6:633. doi: 10.3389/fpls.2015.00633 (PMC4534801; doi:10.3389/fpls.2015.00633)
Supplement: Supplementary file 7 [file Presentation4.PPTX]

## Slide 1
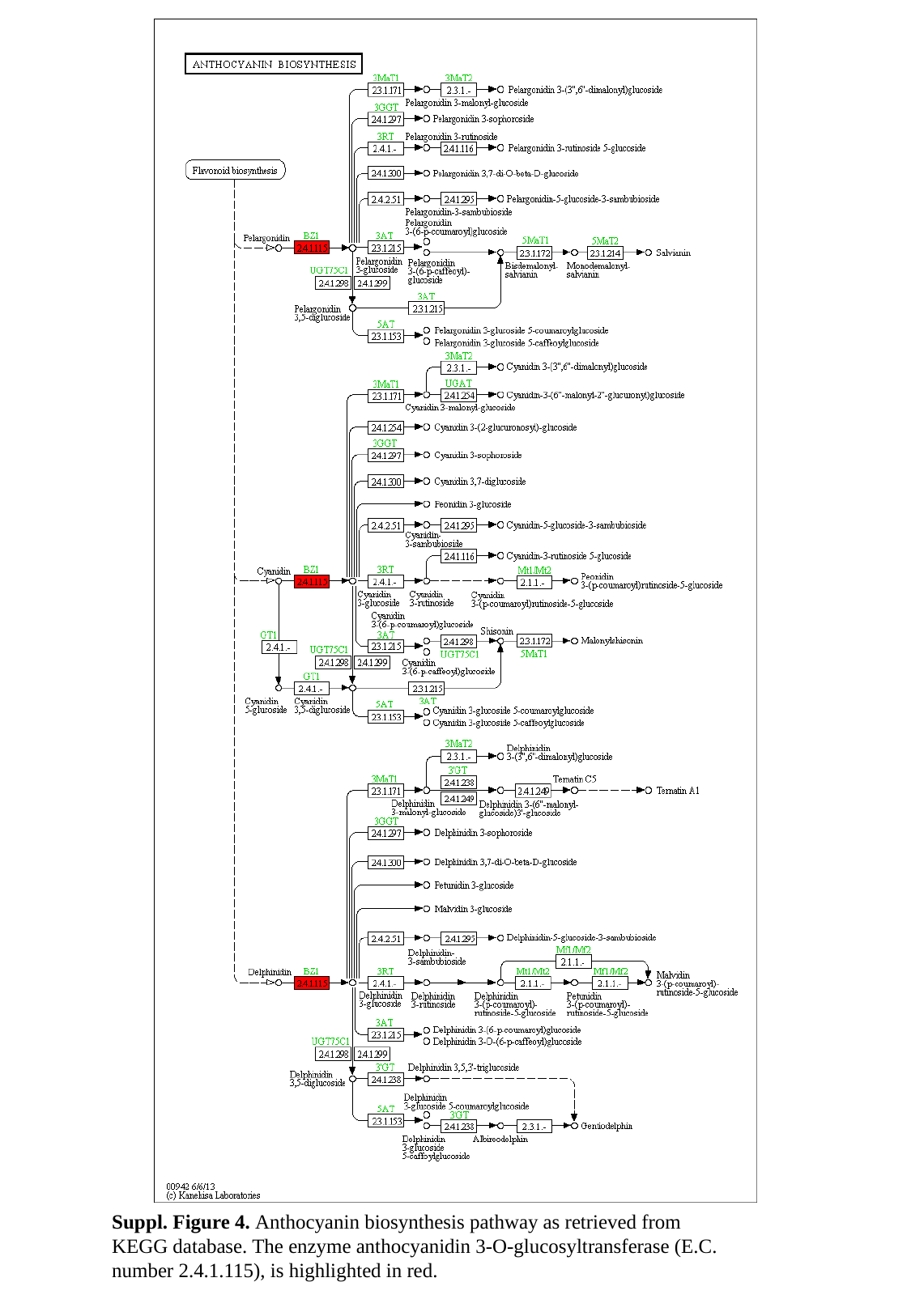

Suppl. Figure 4. Anthocyanin biosynthesis pathway as retrieved from KEGG database. The enzyme anthocyanidin 3-O-glucosyltransferase (E.C. number 2.4.1.115), is highlighted in red.
