# Supplementary material for: Transcriptional profiling unravels potential metabolic activities of the olive leaf non-glandular trichome
Source: Front Plant Sci. 2015 Aug 13;6:633. doi: 10.3389/fpls.2015.00633 (PMC4534801; doi:10.3389/fpls.2015.00633)
Supplement: Supplementary file 8 [file Presentation5.PPTX]

## Slide 1
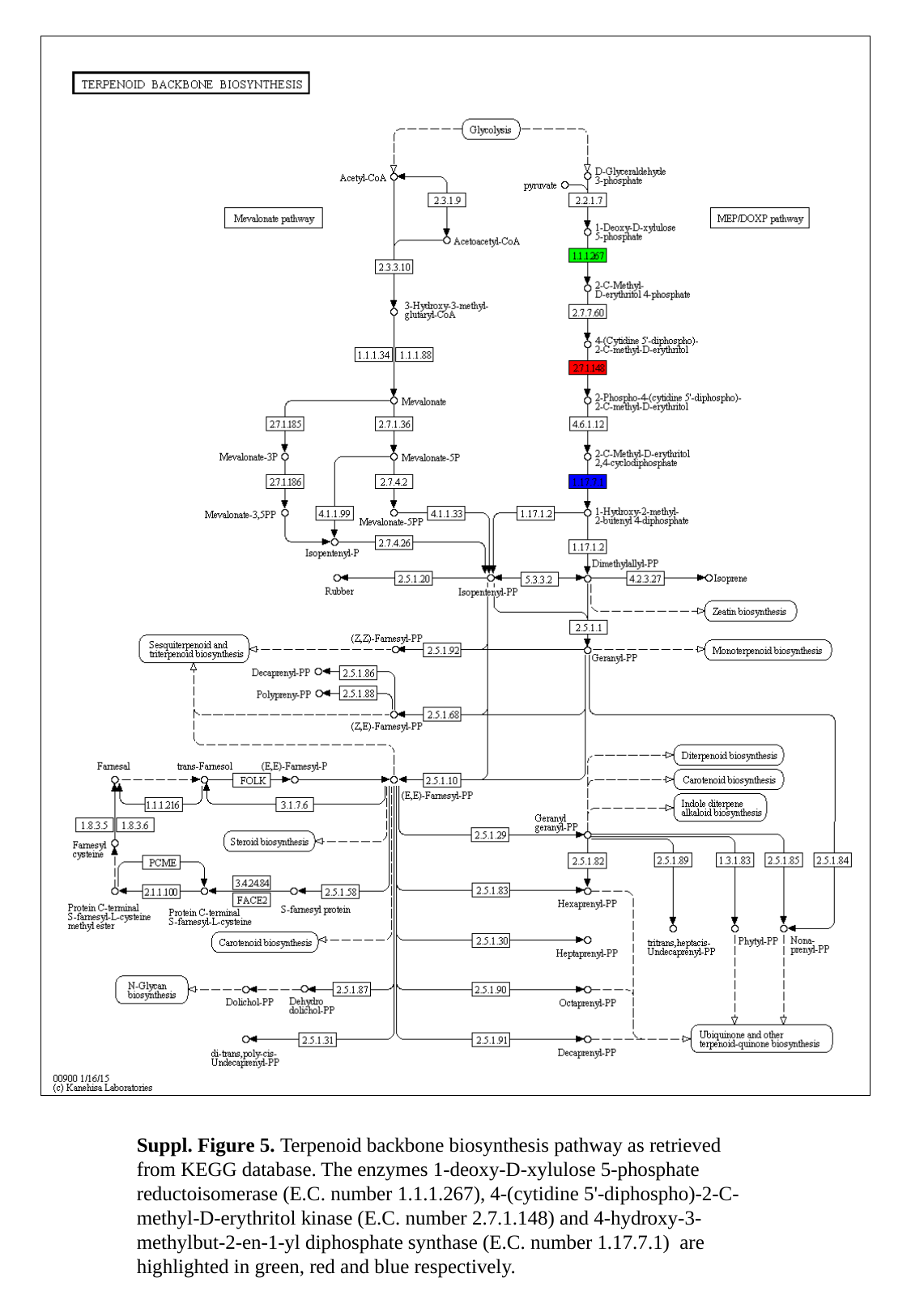

Suppl. Figure 5. Terpenoid backbone biosynthesis pathway as retrieved from KEGG database. The enzymes 1-deoxy-D-xylulose 5-phosphate reductoisomerase (E.C. number 1.1.1.267), 4-(cytidine 5'-diphospho)-2-C-methyl-D-erythritol kinase (E.C. number 2.7.1.148) and 4-hydroxy-3-methylbut-2-en-1-yl diphosphate synthase (E.C. number 1.17.7.1) are highlighted in green, red and blue respectively.
